# Controlling Drug Partitioning in Individual Protein Condensates through Laser-Induced Microscale Phase Transitions

**DOI:** 10.1021/jacs.4c06688

**Published:** 2024-07-04

**Authors:** Axel Leppert, Jianhui Feng, Vaida Railaite, Tomas Bohn Pessatti, Carmine P. Cerrato, Cecilia Mörman, Hannah Osterholz, David P. Lane, Filipe R. N. C. Maia, Markus B. Linder, Anna Rising, Michael Landreh

**Affiliations:** † Department of Cell and Molecular Biology, Uppsala University, S-75124 Uppsala, Sweden; ‡ Department of Microbiology, Tumor and Cell Biology, Karolinska Institutet, S-17165 Solna, Sweden; § Bioproducts and Biosystems, Aalto University, Fi-00076 Aalto, Espoo, Finland; ∥ Department of Anatomy Physiology and Biochemistry, Swedish University of Agricultural Sciences, S-75007 Uppsala, Sweden; ⊥ Department of Biosciences and Nutrition, Karolinska Institutet, S-14157 Huddinge, Sweden; # Department of Biology and Chemistry, Paul Scherrer Institute, 5232 Villingen, Switzerland

## Abstract

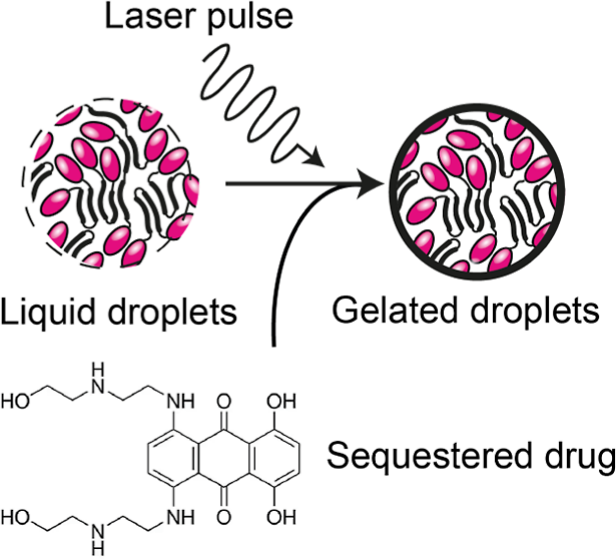

Gelation of protein condensates formed by liquid–liquid phase separation occurs in a wide range of biological contexts, from the assembly of biomaterials to the formation of fibrillar aggregates, and is therefore of interest for biomedical applications. Soluble-to-gel (sol–gel) transitions are controlled through macroscopic processes such as changes in temperature or buffer composition, resulting in bulk conversion of liquid droplets into microgels within minutes to hours. Using microscopy and mass spectrometry, we show that condensates of an engineered mini-spidroin (NT2repCT^YF^) undergo a spontaneous sol–gel transition resulting in the loss of exchange of proteins between the soluble and the condensed phase. This feature enables us to specifically trap a silk-domain-tagged target protein in the spidroin microgels. Surprisingly, laser pulses trigger near-instant gelation. By loading the condensates with fluorescent dyes or drugs, we can control the wavelength at which gelation is triggered. Fluorescence microscopy reveals that laser-induced gelation significantly further increases the partitioning of the fluorescent molecules into the condensates. In summary, our findings demonstrate direct control of phase transitions in individual condensates, opening new avenues for functional and structural characterization.

## Introduction

Liquid–liquid phase separation (LLPS) is a widespread phenomenon in nature, connecting physics, chemistry, biology, and material science.^1,2^ The regulated assembly of proteins through LLPS is an important mechanism for the formation of biologically active condensates within living cells but also the basis for solid biomolecular structures like squid beak and spider silk, shifting the physical state of the protein assembly.^3−5^ During LLPS, proteins separate from a uniform single phase into a protein-rich, condensed phase and a protein-scarce, dilute phase. Depending on molecular interactions and external conditions, the material properties of protein droplets dynamically change and range from liquid-like to dynamically arrested gel- or glass-like.^6,7^ However, many proteins that form biomolecular condensates can undergo multiple phase transitions where the liquid-like properties are lost and the droplets exhibit gel-like features, such as the inability to fuse, increased chemical stability, and nonspherical shapes.^8^ In several human diseases, proteins that have a strong propensity for β-sheet formation, like fused in sarcoma, tau, and α-synuclein, can be assembled into liquid droplets, which can protect them from aggregation.^9,10^ In some instances, these droplets undergo sol–gel transitions as an intermediate step in the conversion from liquid droplets to fibrils.^11−13^ In other instances, phase transitions are of functional importance. By adding phase separation-promoting sequence tags, Wei et al. were able to assemble functional organelles with colocalized enzymes in bacteria.^14^ During spider silk spinning, protein molecules called spidroins are assembled via LLPS, which in turn prepares them to be converted into a solid fiber.^4,15−18^ Such controlled phase transitions are of importance in biomedical engineering, most notably in the form of silk-based particles that hold great promise as drug carriers. The repeat domains of spidroins have an inherent ability to aggregate under a variety of solution conditions and can be assembled with microfluidics into microspheres with stable β-sheet-rich structures.^19,20^ Mixing denatured repeat domains with small-molecule drugs allows the formation of highly stable drug-loaded particles in the nanometer size range.^21,22^ Modification of the repeat sequence additionally allows tuning of the encapsulation efficiency for molecules of interest. Silk-based nanoparticles have good biocompatibility^23^ and have been employed successfully for intracellular delivery as well as extracellular slow release of anticancer compounds in cell culture models.^24,25^

Recently, the inherent ability of mini-spidroins to undergo LLPS has moved into focus to generate particles via phase separation.^26,27^ The liquid-like properties of condensates potentially allow improved control of particle loading, shape, and size range, and additional phase transitions are required to convert the chemically labile condensates into stable microgels.^28,29^ Such phase transitions are usually triggered on a large number of droplets by exposing them to an external stimulus, like changes in temperature, ionic strength, pH, or cofactors.^8,28^ Control of individual droplet transitions is achieved in microfluidics-based set ups (for example, see refs (30–32)). As a result, spatiotemporal control of droplet gelation remains challenging. However, one way to locally steer phase transitions in proteins is the use of focused light, as exemplified using cryptochrome domains to engineer proteins that exhibit light-induced LLPS.^33^ Here, we demonstrate that laser pulses can be used to control sol–gel transitions in mini-spidroins that undergo functional phase transitions as part of the spinning process. By targeting single spidroin droplets with laser pulses at micrometer resolution, we accelerate the gelation process in single droplets from hours to seconds using fluorescent probes with specific absorption properties. We show that gelation enhances the affinity for spidroin-tagged proteins and small molecules. Our findings demonstrate control of sol–gel transitions down to the microscale, which opens new possibilities to study the molecular mechanism of phase transitions, as well as develop laser-inducible gels that trap proteins or drugs for microscale applications.

## Results and Discussion

### Fluorescence Spectroscopy and Mass Spectrometry Show NT2RepCT^YF^ Microgel Formation

As a test case for functional, controllable phase transitions, we turned to the designed mini-spidroin NT2RepCT, which features a conserved three-domain architecture with nonrepetitive N-terminal (NT) and C-terminal (CT) domains encapsulating a central region with two alanine- and glycine-rich repeat sequences.^34,35^ Importantly, NT2RepCT, as well as the isolated NT, can be converted into β-sheet-rich hydrogels through incubation at elevated temperatures and high concentrations.^36,37^ Spidroin hydrogels have been used for cell culture and the release of therapeutic biologicals.^21,37^ Macroscopic liquid-to-solid transitions of NT2RepCT can be triggered by pH and shear force, as well as changes in salt concentrations or temperature, making it a suitable system to study sol–gel transitions at the microscale.^35,37^ We selected the NT2RepCT^YF^ variant, where all Tyr residues in the repetitive region are exchanged to Phe, which exhibits robust LLPS without affecting its ability to be spun into fibers.^38,39^ These observations can be explained by a preference for spherical assembly due to increased contributions from π stacking.^40^

**Figure 1:**
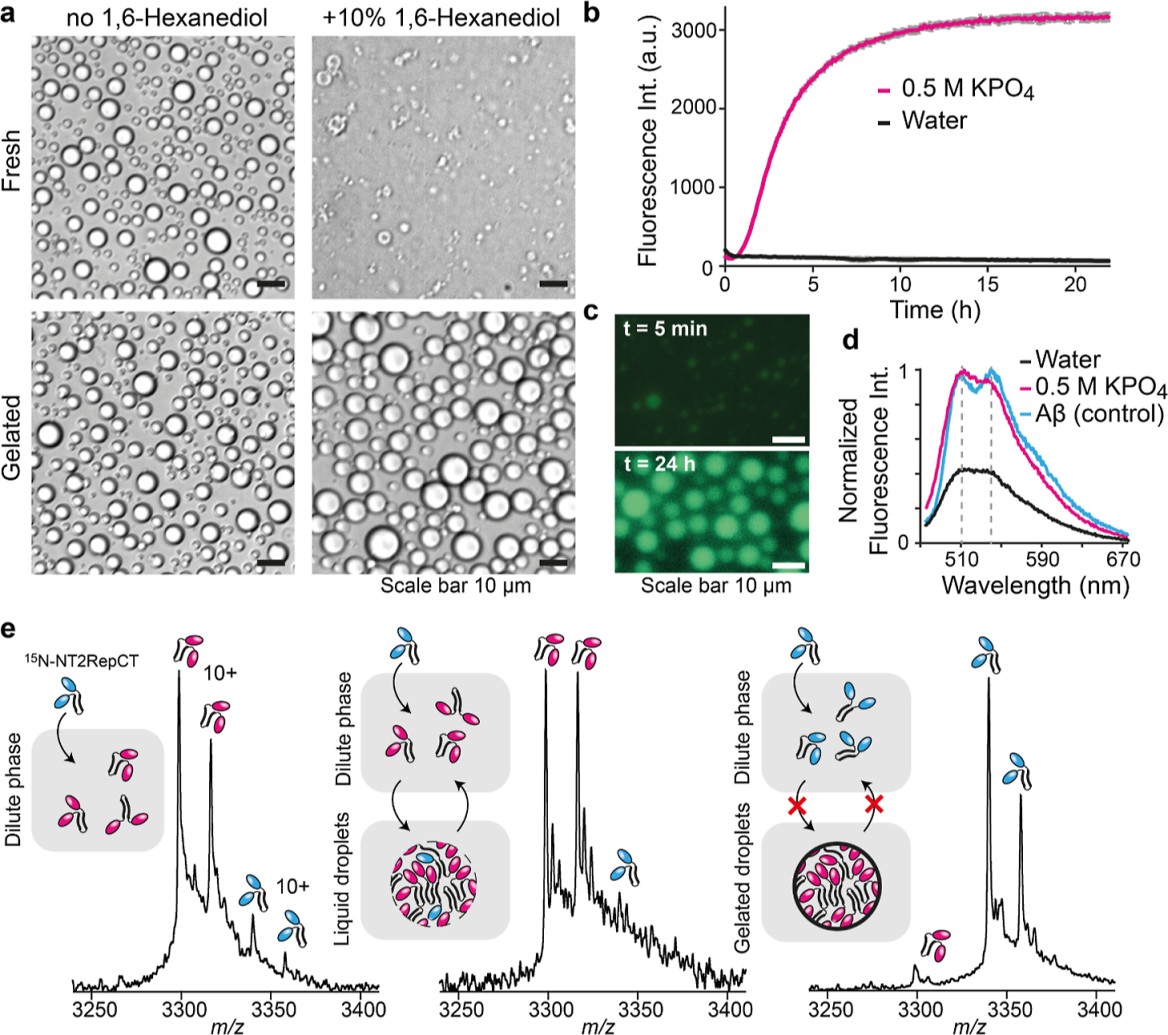
Gelation of NT2RepCT^YF^ droplets. (a) Bright-field images show that freshly formed NT2RepCT^YF^ droplets dissolve upon addition of 1,6-hexanediol (top row) but become 1,6-hexanediol-resistant upon gelation (bottom row). Scale bars are 10 μm. (b) NT2RepCT^YF^ droplets exhibit an increase in ThT fluorescence during incubation under LLPS conditions (0.5 M KPO_4_, pH 8). Data are presented as mean ± standard deviation (gray) of 4 replicates. (c) Fluorescence microscopy shows weak ThT staining of the droplets after 5 min, as well as strong ThT staining at the end point of gelation. Scale bars are 10 μm. (d) The emission spectrum of NT2RepCT^YF^ droplets after incubation (pink curve) stained with pFTAA shows the characteristic maxima indicating β-sheet formation. The spectra of NT2RepCT^YF^ in water and of Aβ_42_ fibrils (positive control) are shown in black and blue, respectively. (e) An nMS assay shows the sol–gel transition of NT2RepCT^YF^ droplets. Left: mass spectrum of 100 μM NT2RepCT^YF^ with 10 μM ^15^N-labeled NT2RepCT^YF^ under non-droplet conditions (100 mM ammonium acetate). Middle: The mass spectrum of ^15^N-NT2RepCT^YF^ added to fresh NT2RepCT^YF^ droplets shows the rapid equilibration of the labeled and unlabeled protein in the dilute phase. Right: mass spectra of ^15^N-NT2RepCT^YF^ added to gelated NT2RepCT^YF^ droplets show that the labeled protein remains in the dilute phase. The double peaks stem from formyl-methionine cleavage in the *E. coli* expression host. Unlabeled spidroins in the schematics are rendered in pink, and ^15^N-labeled spidroins in blue. Full mass spectra are shown in Figure S1d.

Combining the conditions for droplet formation and gelation,^16,36,38^ we incubated NT2RepCT^YF^ at a concentration of 25 μM in 0.5 M KPO_4_, pH 8, overnight. We observed a moderate increase in droplet size, in line with previous studies,^38^ but no other morphological changes compared to fresh droplets. However, upon addition of 10% 1,6-hexanediol, a potent LLPS disruptor,^35^ fresh droplets readily dissolved, leaving some amorphous aggregates, whereas the incubated droplets remained unaffected, indicating gelation (Figure 1a). Since both spun and gelated NT2RepCT is rich in β-sheet structures,^35,37^ we tested whether this also is the case for gelated NT2RepCT^YF^ droplets. Indeed, incubation of fresh droplets with the dye Thioflavin T (ThT) yielded an increase in fluorescence over 24 h (Figure 1b). Fluorescence microscopy showed weak ThT staining after 5 min, indicating that the dye is recruited into the droplets, as well as strong uniform staining after 24 h (Figure 1c). Since ThT fluorescence is sensitive to changes in viscosity, we additionally probed the conformation of NT2RepCT^YF^ in droplets using the dye pFTAA, which exhibits a specific, characteristic two-maximum emission spectrum when bound to β-sheet structures.^41,42^ We found that gelated droplets exhibit a two-maximum spectrum comparable to that of the model amyloid Aβ_42_ (Figure 1d). Our fluorescence data suggest that gelation of NT2RepCT^YF^ droplets may involve an increase in β-sheet content, although other structural changes cannot be excluded. Together, the resistance to 1,6-hexanediol and an increase in β-sheet-specific dye fluorescence indicate the formation of macroscopic dynamically arrested spidroin assemblies during gelation. To test this possibility, we developed a native mass spectrometry (nMS) assay based on the assumption that proteins undergo constant exchange between the condensed and the dilute phase in liquid but not in gelated droplets. Briefly, we added ^15^N-labeled NT2RepCT^YF^ to unlabeled NT2RepCT^YF^ and determined the ratio of both proteins in the dilute phase (the supernatant) with nMS. Next ^15^N-labeled protein was added to 100 μM unlabeled and already phase-separated protein at a concentration of 10 μM, below the threshold for droplet formation, resulting in a 10:1 ratio of unlabeled to labeled NT2RepCT^YF^ in the total protein population (Figures 1e, left and S1a–c). When ^15^N-NT2RepCT^YF^ was added to freshly formed unlabeled droplets, a similar ratio of unlabeled to labeled protein was detected in the dilute phase, indicating that the labeled protein rapidly equilibrated with the unlabeled protein across the condensed and dilute phases (Figure 1e, middle). Upon addition of ^15^N-NT2RepCT^YF^ to unlabeled gelated droplets, virtually only labeled protein was detected in the dilute phase, suggesting that it is excluded from the condensed phase (Figure 1e, right). We conclude that the incubation of NT2RepCT^YF^ under LLPS conditions converts liquid droplets to spherical microgels, which is potentially accompanied by an overall increase in β-sheet content.

### NT Domain Facilitates Selective Protein Incorporation into Gelated Droplets

**Figure 2:**
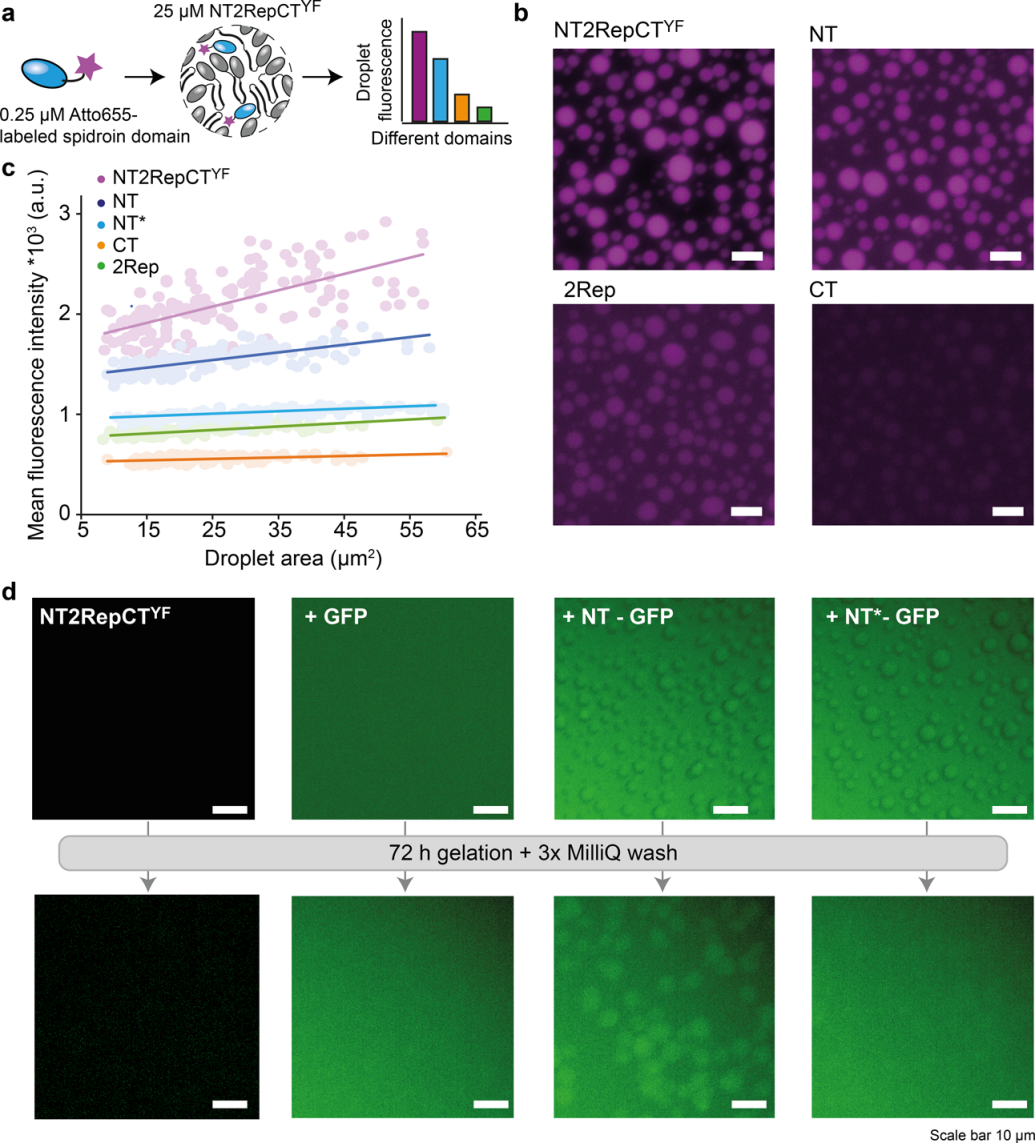
NT domain enables recruitment and trapping of a target protein in NT2RepCT^YF^ droplets. (a) Principle of the recruitment assay. Fluorescently labeled spidroin domains are incorporated into unlabeled spidroin condensates. Droplet fluorescence as a function of the droplet area is quantified by microscopy. (b) Fluorescence microscopy images of fresh condensates formed by 25 μM NT2RepCT^YF^ in the presence of Atto655-labeled spidroin domains show different fluorescence intensities, indicating differences in recruitment efficiency. Scale bars are 5 μm. (c) Quantification of spidroin domain recruitment. Full-length mini-spidroins and NT are efficiently incorporated into droplets, whereas recruitment of the repeat domain (2Rep) or CT is less efficient. Recruitment of the nongelating NT^D40K/K65D^ (NT*) variant is notably lower than that of the NT. (d) Recruitment and trapping of GFP. Fluorescence microscopy images of fresh NT2RepCT^YF^ droplets incubated (from left to right) without GFP, with GFP, with NT-GFP, and with NT*-GFP. NT-GFP and NT*-GFP colocalize with the droplets. Bottom row: the same samples were imaged after gelation by 72 h incubation at 37 °C, followed by three washes with water. Only NT-GFP remains colocalized with the droplets. Scale bars are 10 μm.

As indicated by the MS data, gelated droplets exhibit little to no protein exchange with the surrounding dilute phase. Such rapid gelation could be employed to trap proteins inside the droplets. We therefore investigated which sequence features promote uptake of proteins into NT2RepCT^YF^ droplets. For this purpose, we produced individual domains (NT, 2Rep, and CT) recombinantly and labeled each with Atto655 dye. We then mixed 0.25 μM labeled protein with 25 μM unlabeled NT2RepCT^YF^ and monitored the uptake using fluorescence microscopy upon droplet formation (Figure 2a). Using Atto655-labeled full-length NT2RepCT^YF^ as the standard, we find that the NT is most efficiently incorporated into droplets, followed by 2Rep, whereas CT recruitment is barely detectable. We quantified uptake by measuring the fluorescence intensity as a function of the droplet area (Figure 2b). The results clearly indicate preferential recruitment of the NT. We speculate that the NT may form weak dimers with the free NT domains of NT2RepCT^YF^. To test this hypothesis, we added Atto-labeled NT^D40K/K65D^ (referred to as NT*), a charge-swapped variant that does not dimerize or form hydrogels.^37,43^ We found that NT* uptake is far lower than that of the wild-type NT, suggesting that NT interactions are an efficient way to control recruitment into spidroin droplets (Figure 2c).

Next, we assessed whether gelation could be employed to trap NT-tagged proteins inside the condensates. We incubated NT2RepCT^YF^ droplets with either untagged GFP, NT-GFP, or NT*-GFP (Figure 2d) and monitored uptake via fluorescence microscopy. Addition of NT-GFP and NT*-GFP resulted in green-fluorescent droplets, while untagged GFP was not incorporated to a detectable degree. We then incubated the droplets at 37 °C for 72 h to induce gelation, followed by three 1 h washes with Milli-Q, and imaged the droplets again. Strikingly, only NT-GFP remained in the gelated droplets after the washing step (Figure 2d). These data suggest that tagging proteins with the NT, which incidentally is a potent expression tag,^43−45^ enables us to load condensates with a specific protein of interest and subsequently immobilize it using controlled gelation of the spidroins.

These findings show that proteins can retain their native fold inside gelated spidroin droplets, as evident from the GFP data, which suggests that such droplets could be functionalized by incorporating enzymes. Furthermore, recruitment via a folded domain that undergoes aggregation results in specific retention of the tagged protein, as shown by the fact that NT*-GFP is not retained inside the droplets after gelation. We speculate that tagging other phase-separating proteins with the NT could be used to drive the recruitment of a protein of interest to the corresponding droplets.

### Laser Pulses Induce Sol–Gel Transitions of Individual NT2RepCT^YF^ Droplets

**Figure 3:**
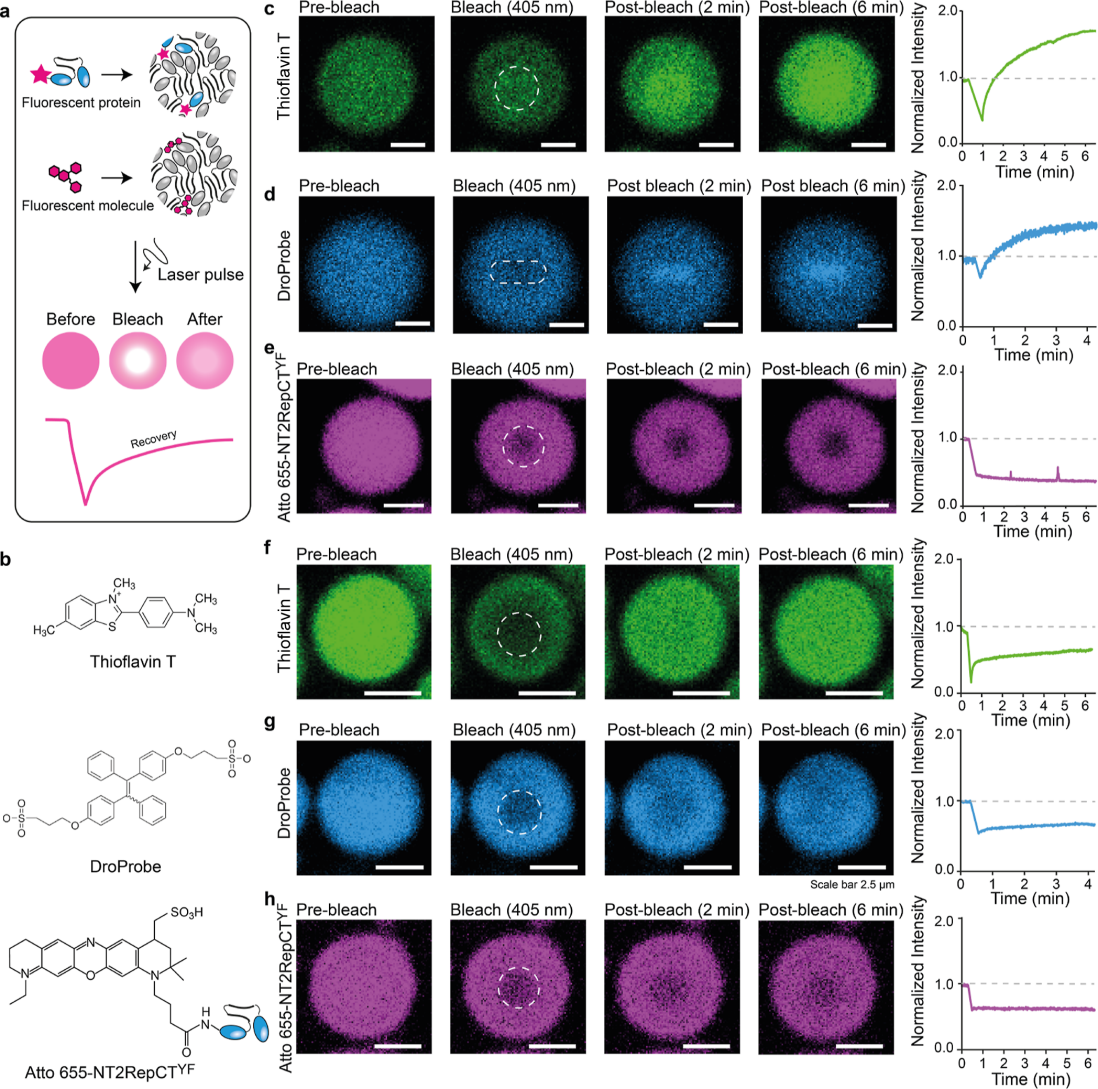
Laser-induced sol–gel transitions in individual fresh, but not gelated, NT2RepCT^YF^ droplets. (a) Principle of FRAP. Spidroin droplets incorporating either fluorescently labeled NT2RepCT^YF^ or fluorescent small-molecule dyes are subjected to bleaching with short laser pulses. If fluorescent molecules can move freely in the droplet, fluorescence in the bleached spot recovers in a time-dependent manner. (b) Structures of the fluorescent probes used in this study. (c) The dye ThT shows an increase in fluorescence after FRAP, which spreads from the bleached area (dashed circle) to the whole droplet. (d) The viscosity dye DroProbe also exhibits increased fluorescence after FRAP, while the bleached area initially retains its rectangular shape. (e) FRAP of NT2RepCT^YF^ droplets containing 0.25 μM Atto655-labeled protein shows no recovery in the bleached area (dashed circle). (f–h) Gelated droplets show slow, partial recovery of (f) ThT and (g) DroProbe fluorescence and (h) no recovery of Atto655-NT2RepCT^YF^ fluorescence after photobleaching. Time-dependent fluorescence intensity plots for the center of the photobleached area are shown to the right of each series. Note that each plot refers to the droplet shown on the left. See Figure S2a for representative errors. Scale bars are 5 μm in (c–e) and 2.5 μm in (f–h).

The most-studied phase transitions occur in neurodegeneration-associated proteins, such as FUS, tau, and α-synuclein. Here, amyloidogenic segments in unstructured protein regions inside the dynamic phase-separated assemblies make stochastic contacts with each other, eventually nucleating fibrillar structures over time.^46,47^ In NT2RepCT, on the other hand, the most amyloidogenic regions are concealed in the folded terminal domains which readily change their structures following external cues.^48^ To better understand how the metastability of spidroins affects gelation, we turned to fluorescence recovery after photobleaching (FRAP) coupled with microscopy, an established tool to assess the dynamics of protein condensates, which we applied to the fluorescent small molecules ThT and DroProbe as well as Atto655-labeled proteins (Figure 3a,b).^49,50^ As a first step, we sought to clarify the mobility of ThT in fresh droplets. To our surprise, we found that a 20 s pulse with a laser wavelength of 405 nm at 25% laser power caused an immediate increase in ThT fluorescence intensity, which overshot the prebleaching fluorescence by 1.3-fold (Figure S2a). When the laser power was raised to 100%, the overshoot increased to approximately 1.7-fold with a narrow standard deviation (Figure S2a). Microscopy revealed that the ThT fluorescence increase starts in the bleached spot and spreads out within 5 min (Figure 3c). For comparison, we performed FRAP with full-length human tau (htau), an established amyloidogenic phase-separating protein.^11^ Even at high laser energies, ThT displayed recovery with no overshoot (Figure S2b). To test whether this behavior is specific for ThT, we repeated the experiment using the viscosity-sensitive DroProbe reagent which absorbs very little at 405 nm^51^ and additionally selected a rectangle as the laser-exposed region to test whether the shape would be retained. As with ThT, DroProbe rapidly diffused back into the bleached area, which remained rectangular for >2 min and exhibited a 1.5-fold overshoot in fluorescence postbleach (Figure 3d). The same feature was observed for droplets composed of NT2Rep with no CT (Figure S2c). We then asked whether the protein itself also remained mobile after the laser pulse. We therefore assembled fresh droplets containing 0.25 μM Atto655-labeled NT2RepCT^YF^ and performed FRAP at a laser wavelength of 405 nm at a maximum laser power. The Atto655 fluorescence in the bleached region of the droplet did not recover, suggesting a nonliquid state. Yet, neighboring unbleached droplets continued to fuse, as expected for liquid condensates (Figure S2d). Att655-labeled htau, on the other hand, displayed normal recovery when subjected to the same FRAP experiment (Figure S2b). Taken together, these data suggest that the spidroins in the bleached area convert into a more viscous phase, indicating gelation. To confirm this interpretation, we repeated the experiments with already gelated droplets in the absence of any light source during incubation at 25 °C for 72 h. ThT and DroProbe exhibited stronger fluorescence than in fresh droplets from the start but did not show a fluorescence overshoot after slowly diffusing back into the bleached regions (Figure 3f,g). As expected, Atto655-labeled NT2RepCT^YF^ did not diffuse back into the bleached spot (Figure 3h), strongly suggesting that once the droplets have undergone a sol–gel transition, the laser-induced fluorescence overshoot is abolished.

The FRAP data suggest that laser pulses can greatly accelerate the gelation of dye-loaded fresh spidroin droplets, reducing the time from hours to seconds. This finding led us to ask whether the same effect could be elicited without the dye. Interestingly, amyloid-like fibrils absorb light at wavelengths between λ 360 and 700 nm and exhibit red-shifted fluorescence in the visible and near-IR region.^52−54^ The origin of the phenomenon, which is particularly prominent in silk fibers,^55^ is not clear, and multiple explanations have been put forward, including quantum confinement effects, electron delocalization, and charge transport.^56,57^ LLPS of spidroins promotes self-assembly and β-sheet fibrillation, although the β-sheet content may differ depending on the spidroin sequences used.^4,16,18,38^ We therefore tested whether spidroin droplets could interact with visible light in a manner similar to that of silk fibers. Indeed, we found that excitation at λ 405 nm and λ 561 nm (blue and green light, respectively) resulted in red-shifted fluorescence of fresh droplets, which became more intense upon gelation (Figure S3a). htau droplets, on the other hand, exhibited pronounced fluorescence only at λ 405 nm excitation (Figure S3a), similar to htau fibrils.^58^ The data indicate that some protein condensates, such as amyloid-like fibers, may have protein-specific fluorescence properties. Importantly, the red-shifted fluorescence confirms that spidroin droplets can absorb visible light and that some of the energy is dissipated in other ways than photon emission. It is therefore likely that the absorption of high-energy laser pulses of the same wavelength may help to overcome the energy barrier for gelation, for example, through heating, since a temperature increase of 4–7 K is enough to trigger the assembly of the NT into hydrogels.^37^ We performed FRAP experiments by bleaching dye-free fresh droplets at a laser wavelength of 405 nm. We observed a sudden increase of the intrinsic fluorescence, albeit less pronounced than for ThT and DroProbe (Figure S3b). These data suggest that laser pulses can elicit a gelation process similar to incubation at elevated temperatures. Our findings open the possibility of using spidroin domains to engineer protein condensates with laser-inducible sol–gel transitions.

### Laser-Induced Sol–Gel Transitions Can Be Tuned by Fluorescent Drugs

**Figure 4:**
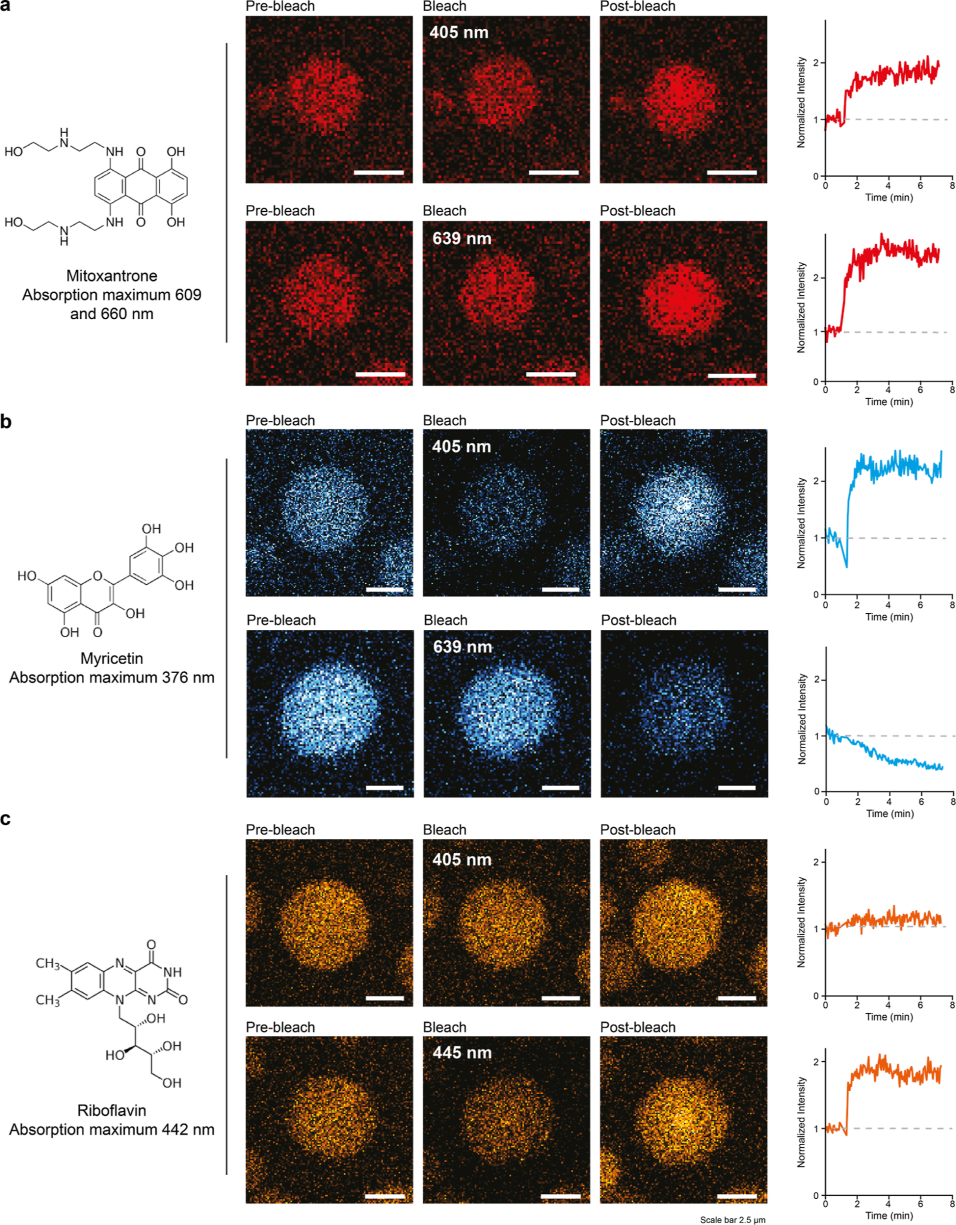
Laser-induced sol–gel transitions are tuned by fluorescent drugs. (a) Fluorescence microscopy images of NT2RepCT^YF^ droplets in the presence of 25 μM mitoxantrone show red fluorescence, indicating drug recruitment into the droplets. Top row: photobleaching at 405 nm results in a 2-fold fluorescence overshoot. Bottom row: photobleaching at 639 nm, near the absorbance maxima of 609 and 660 nm, induces a strong increase in mitoxantrone fluorescence, with a near 3-fold overshoot in fluorescence intensity. (b) Top row: photobleaching of NT2RepCT^YF^ droplets containing myricetin causes bleaching and a fluorescence overshoot when bleached at 405 nm. Bottom row: no overshoot after bleaching at 639 nm. The absorbance maximum of myricetin is 376 nm. (c) Top row: NT2RepCT^YF^ droplets containing riboflavin show a very minor fluorescence overshoot when photobleached at a laser wavelength of 405 nm. Bottom row: photobleaching at 445 nm, near the absorbance maximum of 442 nm, results in a fluorescence overshoot indicating gelation. Scale bars are 2.5 μm.

Protein condensates can sequester fluorescent drugs by engaging their aromatic moieties in nonspecific π–π and π–cation interactions.^59^ Importantly, sequestration can reduce the efficacy of these compounds by preventing them from reaching their therapeutic targets, but how such interactions are affected by phase transitions within the condensate is not known. Inducing gelation at the microscale raises the possibility of investigating whether small-molecule recruitment and laser-induced sol–gel transitions are related. We selected mitoxantrone, an anthracenedione used to treat acute myeloid leukemia and multiple sclerosis and which partitions into nuclear condensates in vitro.^59^ Mitoxantrone has a broad absorption range from 490 to 693 nm, with a pronounced maximum of 609 and 660 nm. Recruitment of mitoxantrone into spidroin condensates was confirmed by monitoring the intrinsic fluorescence of mitoxantrone in fresh NT2RepCT^YF^ droplets (Figure 4a). We then performed photobleaching with a 20 s maximum energy laser pulse with a 405 nm wavelength on an individual droplet. We observed a 1.5-fold fluorescence overshoot which increased to 2-fold after 5 min (Figure 4a). We then repeated the experiment using a wavelength of 639 nm, near the absorbance maxima for mitoxantrone at 609 and 660 nm. Bleaching at this higher wavelength caused a 2-fold overshoot in fluorescence already at the end of the pulse, which subsequently increased to 3-fold within 2 min. Unlike ThT and DroProbe, mitoxantrone fluorescence, which stems from the rigid anthracene moiety, is independent of the chemical environment. Therefore, the increase likely indicates that the gelated droplet has a higher affinity for the drug in comparison to the surrounding liquid droplets. To confirm this interpretation, we performed a double-photobleaching experiment. A fresh, once-bleached droplet was allowed to recover before being bleached a second time. We then measured the resulting change in mitoxantrone fluorescence. While the fluorescence recovery after the second bleach was rapid, the droplet fluorescence did not increase over the original level, indicating that the sol–gel transition induced by the first photobleaching event drives mitoxantrone recruitment. Neighboring droplets exhibited no change in fluorescence, both for NT2RepCT and NT2RepCT^YF^ condensates (Figure S4a,b). To confirm that we are indeed able to manipulate the sol–gel transition of an individual droplet, we tested the droplet stability after the addition of 1,6-hexanediol. Applying our bleaching approach, we first enhanced mitoxantrone partitioning in a single droplet (Figure S4c). We then directly added 10% 1,6-hexanediol to the supernatant, which disrupted the integrity of all condensates except the previously bleached droplet. Similarly, previously bleached droplets were found to be resistant to 10% formic acid (Figure S4d). Z-stack imaging confirmed that mitoxantrone is evenly distributed throughout the droplet (Figure S4e). The preferential mitoxantrone partitioning into the gelated droplets appears to be surprising. nMS reveals only very weak interactions between the NT and mitoxantrone, suggesting that binding to the folded domain is not responsible for recruitment into droplets (Figure S4f). However, it was recently reported that tyrosine residues in the repeat regions of spidroins experience a shift in the chemical environment during phase transitions.^60^ Furthermore, interactions with aromatic residues are responsible for drug partitioning into condensates.^59^ Although modeling structural states of the proteins in gelated droplets is not possible at this stage, we speculate that the aromatic residues in NT2RepCT^YF^ may arrange in a way that increases their affinity for aromatic compounds, possibly by enabling π stacking with mitoxantrone.

These data suggest that the optimum wavelength for laser-induced gelation may be affected by the presence of highly fluorescent drugs and that preferential recruitment of these drugs into gelated droplets can serve as a readout for gelation. To test the hypothesis, we selected two additional fluorescent druglike molecules, myricetin, a flavonoid with antioxidant properties, and riboflavin, also known as vitamin B2. Myricetin, which has an absorbance maximum of 376 nm, was readily recruited into the spidroin droplets. Photobleaching at a laser wavelength of 405 nm induced the characteristic fluorescence overshoot, indicating gelation (Figure 4b). Photobleaching at 639 nm, which resulted in a strong fluorescence overshoot for mitoxantrone, did not increase myricetin fluorescence (Figure 4b). Riboflavin, which has an absorption maximum of 442 nm, was also recruited into fresh droplets (Figure 4c). Almost no fluorescence overshoot (less than 1.2-fold) was observed following photobleaching at 405 nm. Photobleaching at 445 nm, on the other hand, resulted in a 2-fold increase in fluorescence (Figure 4c). For all three compounds, the required wavelength for laser-induced gelation correlates directly with the absorbance maximum of each fluorescent dye. We speculate that the dyes act as “antennae”, absorbing laser energy, which then triggers gelation of the spidroins. The fact that gelation can be induced at 405 nm in the presence of ThT (absorbance maximum 400 nm) or DroProbe (absorbance maximum 390 nm) further supports this hypothesis. Such a mechanism would likely require high local dye concentrations, in line with the preferential partitioning of the fluorescent molecules into droplets (Figures 3 and 4). However, further studies are warranted to clarify the structural basis of laser-induced gelation.

### Sol–Gel Transitions Increase Drug Partitioning into Intracellular Condensates in Bacteria

**Figure 5:**
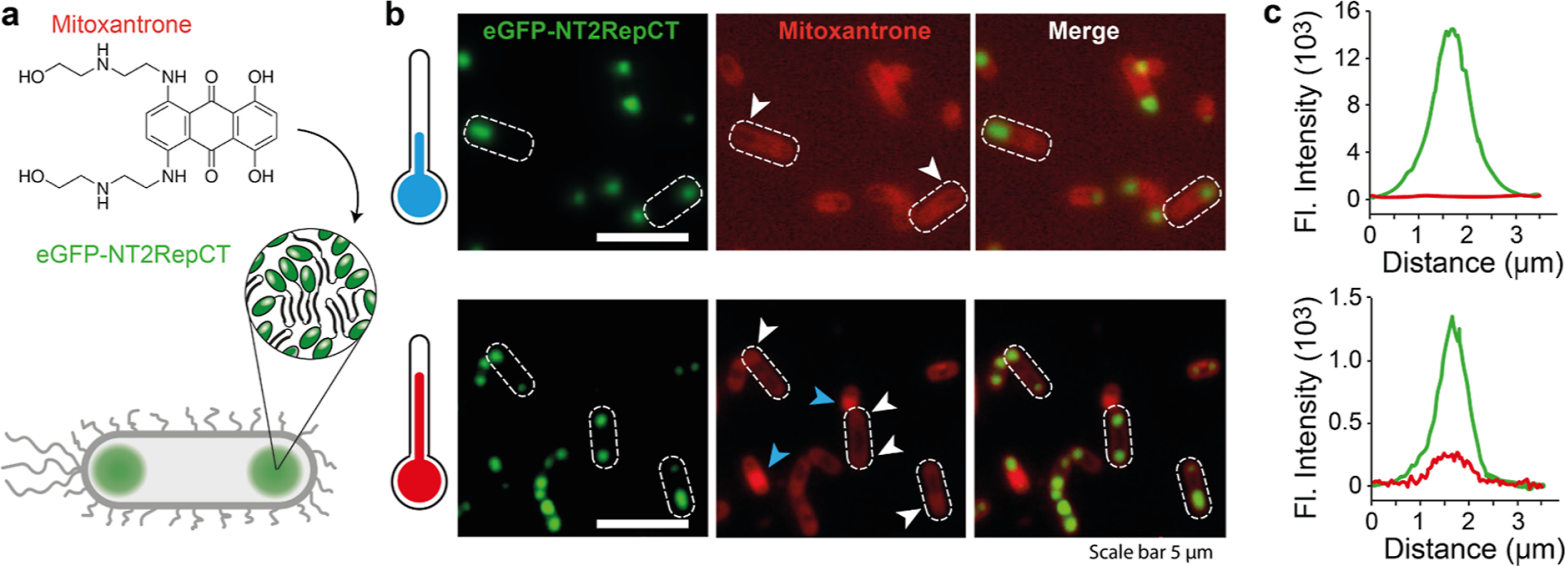
(a) Experimental strategy for mitoxantrone recruitment into intracellular NT2RepCT condensates in *E. coli*. (b) Top row: eGFP-NT2RepCT condensates formed during expression at 18 °C show no colocalization of spidroins (green) and mitoxantrone (red). Instead, mitoxantrone is excluded from the condensates (white arrows). Bottom row: expression at 37 °C results in mitoxantrone concentration at the termini of the bacteria (white arrows). A minor population exhibits strong mitoxantrone fluorescence (blue arrows); we speculate that these cells may be dying due to compound toxicity to *E. coli*. See Figure S5 for phase contrast microscopy overlays of the NT2RepCT-eGFP and mitoxantrone fluorescence images. Scale bars are 5 μm. (c) Fluorescence profiles of individual intracellular condensates show colocalization of mitoxantrone and eGFP-NT2RepCT after 37 °C expression (bottom) but not at 18 °C (top).

Lastly, we asked whether the increase in drug partitioning upon the phase transition also occurs in a cellular environment. For this purpose, we employed eGFP-tagged NT2RepCT, which forms condensates at the poles in *E. coli*.^61^ NT2RepCT droplets display the same increase in mitoxantrone fluorescence upon laser-induced gelation as NT2RepCT^YF^ (Figure S5). Exposing the high laser energies required to induce gelation caused lysis of the cells (Figure S5a). However, we previously observed that expression at 18 °C, but not at 37 °C, yields soluble NT2RepCT which can be purified. FRAP analysis confirmed that spidroin condensates formed at low and high temperatures are liquid and gel-like, respectively (Figure S5b,c). To compare their ability to sequester aromatic compounds in cells, we expressed NT2RepCT at 18 or 37 °C in the presence of mitoxantrone and assessed the colocalization of spidroin and the drug using fluorescence microscopy (Figure 5a). At low expression temperatures, colocalization could not be detected, suggesting that the drug is excluded from the spidroin condensates. At high expression temperature, on the other hand, we observed eGFP-NT2RepCT assemblies which contained mitoxantrone (Figure 5b,c). Taken together, the data show that intracellular spidroin condensates formed at 37 °C recapitulate a key feature of the laser-induced sol–gel transition.

## Conclusions

In this study, we have demonstrated that the designed mini-spidroin NT2RepCT^YF^ readily undergoes a sol–gel transition following LLPS, which can be accelerated dramatically by using laser pulses to allow gelation of individual droplets. We use this approach to show that the resulting microgels have an increased affinity for the antineoplastic compound mitoxantrone both in vitro and in live bacteria. Micromanipulation of protein condensates is a useful tool to study their structure and function in the cellular context.^33^ The fact that mini-spidroin droplets can be gelated by laser pulses at the microscale is likely related to the ability of spidroins to convert to a fibrillar form. While the pFTAA fluorescence indicates an increase in β-sheet formation, the possibility that structural changes in the NT or the repeat region mediate gelation remains speculative. Confirming this hypothesis requires structural characterization with single-droplet resolution, which is an emerging area in condensate research.^62^ While the use of laser pulses and fluorescent probes to induce controlled gelation is not directly applicable to other LLPS assemblies than spidroin droplets, it provides a path to other light-controlled sol–gel transitions, for example, through photoactivatable domains that expose fibril-forming sequences when exposed to light of a specific wavelength. Lastly, we speculate that NT2RepCT is particularly sensitive to phase transitions and that other phase-separating proteins may similarly be subject to laser-induced gelation in the presence of suitable fluorescent probes.

## Supporting Information Available

Experimental details including chemicals, sequences, protein expression and purification, protein labeling, droplet formation and maturation, recruitment of protein constructs into droplets, fluorescence emission spectroscopy and ThT kinetics, fluorescence microscopy of small molecules and FRAP, autofluorescence microscopy, live-cell imaging, sample preparation for nMS, nMS, solubility, ThT fluorescence traces, confocal microscopy, photobleaching, and fluorescence microscopy images (PDF)

## Abbreviations

LLPS: liquid–liquid phase separation
nMS: native mass spectrometry
ThT: thioflavin T
FRAP: fluorescence recovery after photobleaching
htau: human tau protein
